# Multiple-Wearable-Sensor-Based Gait Classification and Analysis in Patients with Neurological Disorders

**DOI:** 10.3390/s18103397

**Published:** 2018-10-11

**Authors:** Wei-Chun Hsu, Tommy Sugiarto, Yi-Jia Lin, Fu-Chi Yang, Zheng-Yi Lin, Chi-Tien Sun, Chun-Lung Hsu, Kuan-Nien Chou

**Affiliations:** 1 Graduate Institute of Biomedical Engineering, National Taiwan University of Science and Technology, Taipei 10607, Taiwan; jiajia527@gmail.com; 2 Graduate Institute of Applied Science and Technology, National Taiwan University of Science and Technology, Taipei 10607, Taiwan; d10622817@mail.ntust.edu.tw; 3 Department of Biomedical Engineering, National Defense Medical Center, Taipei 11490, Taiwan; 4 Department of Neurology, Tri-Service General Hospital, National Defense Medical Center, Taipei 11490, Taiwan; Fuji-yang@yahoo.com.tw; 5 Department of Physical Medicine and Rehabilitation, Taipei City Hospital Zhongxing Branch, Datong District, Taipei 10341, Taiwan; DAP52@tpech.gov.tw; 6 Division of Embedded System and SoC Technology, System Integration and Application Department, Information and Communication Research Laboratory, Industrial Technology Research Institute, Hsinchu 31057, Taiwan; ctsun@itri.org.tw (C.-T.S.); clh@itri.org.tw (C.-L.H.); 7 Neurosurgery Department, Tri-Service General Hospital, Taipei 11490, Taiwan

**Keywords:** wearable device, gait analysis, IMU sensors, gait classification, stroke patients, neurological disorders

## Abstract

The aim of this study was to conduct a comprehensive analysis of the placement of multiple wearable sensors for the purpose of analyzing and classifying the gaits of patients with neurological disorders. Seven inertial measurement unit (IMU) sensors were placed at seven locations: the lower back (L5) and both sides of the thigh, distal tibia (shank), and foot. The 20 subjects selected to participate in this study were separated into two groups: stroke patients (11) and patients with neurological disorders other than stroke (brain concussion, spinal injury, or brain hemorrhage) (9). The temporal parameters of gait were calculated using a wearable device, and various features and sensor configurations were examined to establish the ideal accuracy for classifying different groups. A comparison of the various methods and features for classifying the three groups revealed that a combination of time domain and gait temporal feature-based classification with the Multilayer Perceptron (MLP) algorithm outperformed the other methods of feature-based classification. The classification results of different sensor placements revealed that the sensor placed on the shank achieved higher accuracy than the other sensor placements (L5, foot, and thigh). The placement-based classification of the shank sensor achieved 89.13% testing accuracy with the Decision Tree (DT) classifier algorithm. The results of this study indicate that the wearable IMU device is capable of differentiating between the gait patterns of healthy patients, patients with stroke, and patients with other neurological disorders. Moreover, the most favorable results were reported for the classification that used the combination of time domain and gait temporal features as the model input and the shank location for sensor placement.

## 1. Introduction

Wearable devices, such as accelerometers, gyroscopes, or a combination of the two to form inertial measurement unit (IMU) sensors, have been widely used in gait analysis and the monitoring of physical activity. The validation of algorithms and sensor configurations has been performed in some studies, including the validation of gait event detection in systems such as the motion capture system, force platform, and gait mat system by using the golden standard [1,2,3,4,5]. A. Godfrey et al. in 2014 [3] validated low-cost body-worn sensors for assessing gait in healthy young and older people. Their study used an accelerometer placed on the lower back (L5) and their algorithm was validated against the golden standard from the *GaitRite* mat system. Spatiotemporal gait parameters including step time, step length, step velocity, stride time, variability, and asymmetry were calculated and compared with the result from the gait mat. Their result showed that the sensor arrangement and algorithm were valid and reliable for quantifying gait during continuous walking in both younger and older adults. Using the same sensor placement and algorithm, S. Del Din et al. in 2016 [1] applied wearable sensors for Parkinson Disease’s (PD) subjects and healthy older adults. Their study found an excellent agreement for mean step time, stance time, step length, and step velocity for the older adults group, while for PD groups, agreement was found for step time, stance time, and step velocity. The efficacy of using an accelerometer placed on the distal tibia to determine the Initial Contact and Final Contact gait event was examined by J. Sinclair et al. in 2013 [4]. Compared with the golden standard from the Kitsler Force platform, they found a strong correlation in the duration of stance phase between the two methods and suggest that a shank-mounted accelerometer can be used to accurately and reliably detect gait events.

The application of a wearable device in gait analysis is not limited to gait event detection and the calculation of spatiotemporal parameters, but can be extended to investigate gait stability and variability between normal and pathological gaits [6,7,8,9] and classify various types of gait patterns [10,11,12,13]. Gait stability and harmony in children with Cerebral Palsy (CP) was examined with IMU sensors placed on the lower back (L3–L4) by M. Iosa et al. in 2012 [7,14]. Gait stability can be defined as the ability of the body segment to displace with proper speed so that the upper body oscillation can be minimized by moving the body segment in a coordinated fashion. This parameter can be quantified by measuring the upper body acceleration’s dispersion and smoothness. Meanwhile, gait harmony is the ability of our body to synchronize the symmetric and rhythmic movement by means of inter-limb, intra-limb, and lower–upper coordination. This parameter also can be quantified by upper body acceleration data, in terms of their ratio between odd and even harmonics and the ratio between the gait parameters of two steps from different sides. Those parameters are important in gait analysis because they are related to the stabilization of the body center of mass in order to avoid falls and also of the head in order to steady the optical and vestibular informational flow [15].

Their study used a 10 m walking test with self-selected speed and then calculated the gait stability and gait harmony using the data from the accelerometer and gyroscope; they found a general reduction of gait stability indicated by higher Root Mean Square (RMS) acceleration, acceleration minimal value, and peak-to-peak angular velocity in children with CP [7]. Gait stability in Down syndrome (DS) and Prader–Willi syndrome (PWS) was examined using four IMU sensors placed on the pelvis, trunk, and both shanks by G. Salatino et al. in 2016 [8]. A 10 m walking test with self-selected speed was also used in this study and spatiotemporal gait parameters with RMS acceleration were calculated from the IMU sensors. They found that there were no significance differences among DS, PWS, and normal children for all calculated spatiotemporal parameters; however, acceleration RMS on the pelvic sensors was found to be greater in DS and PWS with respect to normal children.

To classify various types of gait patterns, E. Sejdic et al. in 2016 [10] examined gait signals from accelerometers placed on the lower back to extract time, frequency, and time–frequency domain features in order to differentiate gaits between healthy and clinical populations (PD and peripheral neuropathy subjects). Discriminating gaits related to neurodegenerative diseases such as PD and Huntington’s disease was done in a study by Mannini et al. [12] using different machine learning techniques and IMU sensors placed on both the shank and lower back (between L4 and S2). Both of those studies used the subject’s self-preferred speed for walking trials on a treadmill and walkway, respectively.

Moreover, various types of physical activity have been examined with the use of wearable devices [16,17,18,19,20,21,22]. In 2004, A. V. Rowlands et al. [20] used a triaxial accelerometer placed on the hip to measure the intensity of different activities (moderate and vigorous) in young children and young adults. Meanwhile, in 2009, five different activities (walking, running, cycling, driving, and sports) were classified with a triaxial accelerometer placed on the subject’s waist and different classifiers (Decision Tree and Naïve Bayes) [17].

While also still minimizing the subject’s discomfort, the classification of gait patterns with a wearable device provides more mobility than does the conventional method, which uses complex and expensive motion analysis system equipment. Gait analysis using IMU sensors provides portability, low cost, and flexibility in terms of free-living data collection. Furthermore, gait classification with a wearable device can provide long-term gait assessment, especially in people with disease, by enabling the early detection of gait alteration and by the easiness of gait assessment. 

Therefore, to achieve ideal results in gait pattern classification, various sensor placements and algorithms have been adopted and tested in normal people and people with disease [23,24,25,26]. A. Salarian et al. in 2013 [25] proposed a novel approach to reducing the number of sensing units for wearable gait analysis by continuing to use the double-pendulum model but with a lower number of sensors (only two gyroscope units placed on both sides of the shank). Their approach concluded that it is feasible to reduce the number of sensors needed from four to two to estimate the movement of the thigh from the movement of the shank, which will be useful for ambulatory gait analysis. While a study by A. Salarian et al. was done on PD subjects, L. Carcreff in 2018 [26] conducted a study which compared the spatiotemporal measurement performance of three wearable sensor configurations: shank and thigh, shanks, and the feet. Their placement configuration selection was based on potential applications in children with CP and their result showed that the shank-and-thigh-based sensor configuration was more robust in children with higher levels of disability.

Vienne et al. (2017) [27] published a review on the use of IMU sensors to assess gait quality in patients with neurological disorders. Their study concluded that too many protocols are being adopted in the assessment of gait using IMU sensors, including those for algorithms and sensor placement. From the total of 78 studies examined in that literature review, 16% of them were using more than one sensor with a maximum of seven sensors; half of them (39 studies) were neurological studies; more than 50 studies used the subject’s self-selected speed; and 67 studies were done only in a gait laboratory or hospital, while 9 of them were done in the subject’s home. Consequently, a comparison of a multitude of studies is difficult to conduct, and ascertainment of the optimal sensor placement configuration and classification algorithm is crucial.

However, no study has ever examined IMU sensor placement configuration with different classification algorithms in patients with neurological disorders. Thus, this study aimed to conduct a comprehensive analysis of the placement of multiple wearable sensors (including seven different sensors placements and five different algorithms) for gait analysis and classification in patients with neurological disorders. Features of time domain, gait temporal parameters, and a combination of both were also examined to determine which feature yielded the most favorable classification result. A comparison of the classification of sensors at multiple placements was crucial for reducing the number of sensors used and, hence, the computational load in order to increase the speed of the classification process. Our hypothesis was that sensor placement on the shank would have a better classification result since the gait event detection would be based on sensors placed on the shank.

## 2. Materials and Methods

### 2.1. Subjects

Twenty subjects participated in this study and were divided into two groups: patients with stroke and patients with other neurological disorders. The stroke patients group comprised 11 subjects (mean age = 65.2 ± 13.7 years, height = 162.1 ± 9.66 cm, and weight = 61.8 ± 6.5 kg), while the group of patients with neurological disorders other than stroke comprised the remaining 9 subjects (mean age = 66.4 ± 9.16 years, height = 167.3 ± 9.16 cm, and weight = 63.5 ± 11.1 kg) and consisted of subjects who had undergone surgery for brain tumors and had a spinal injury, brain concussion, or brain hemorrhage. Participants who met the following inclusion criteria were enrolled in this study: age ≥ 18 years; ability to understand simple instructions; ability to walk without any assistive device for at least 15 m. Participants with unstable neurological and functional status were excluded, along with those with comorbidities that would affect gait.

### 2.2. Equipment

Seven wireless IMU sensors from Delsys Trigno^TM^ (Delsys Inc., Boston, MA, USA) were used in this study. The IMU sensors consisted of a triaxial accelerometer, gyroscope, and magnetometer. However, this study only used data from the accelerometer and gyroscope. The triaxial accelerometer can measure acceleration up to ±16 g, and the triaxial gyroscope can measure angular velocity up to 2000°/s. The sampling rate of both the accelerometer and gyroscope was 148 Hz, and the resolution of the analog-to-digital converter was 16 bit. All IMU sensors were connected through wireless communication to the Delsys Sensor Base, which used a USB interface to transfer the data in real time to the PC. All data acquisition procedures were performed using EMGWorks 4.3.1 Acquisition software (Delsys Inc., Boston, MA, USA), and data processing and analysis was performed using Python 3.6 with an Anaconda environment.

### 2.3. Experimental Protocol

**Figure 1:**
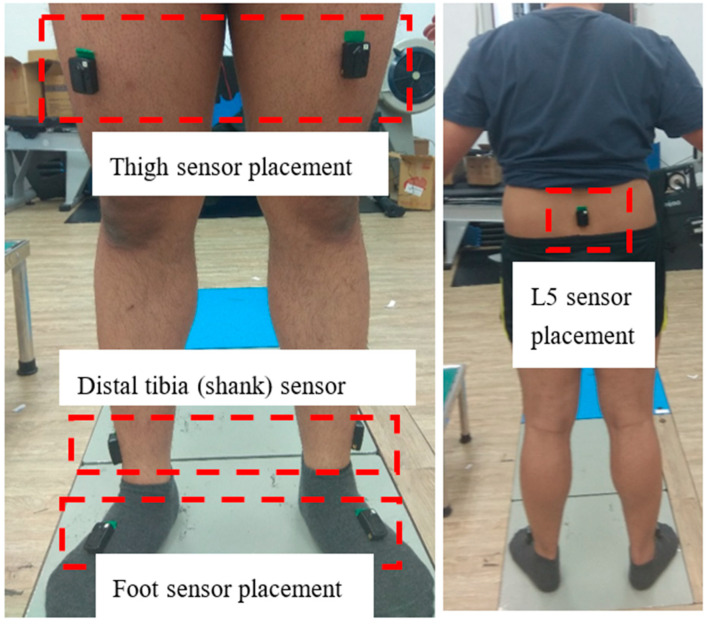
Illustration of the placement of the seven sensors on the subject.

The IMU sensors were placed at seven locations on the subject. The first sensor was placed on the subject’s lower back (L5), and the second and third sensors were placed on the subject’s left and right foot, respectively. Both sides of the subject’s thigh and distal tibia were used for the placement of sensors 4–7. The orientation of the sensor’s axis was set to a mediolateral direction for the *x* axis, a vertical direction for the *y* axis, and an anteroposterior direction for the *z* axis. All of the sensors were secured with tight medical-grade tape to minimize movement artifacts. An illustration of sensor placement is shown in Figure 1.

After all the sensors had been placed, the subject was asked to participate in a level walking trial at their selected speed. One level walking trial included the forward and return directions of a distance of approximately 12 m. Each subject was required to complete six successful trials. All IMU sensor data were collected and saved on the PC for further analysis. In order to avoid the effect of the turn-around period, the return direction walk was excluded and only the first two strides in the forward direction (one right stride and one left stride) were used for data analysis.

Data for the stroke group and the group with other neurological disorders were collected at a hospital, whereas data for the healthy adult group were collected in the gait laboratory.

### 2.4. Data Processing

The triaxial accelerometer and gyroscope data from all seven sensors were extracted and exported to a .csv format file for further processing. Triaxial accelerometer data were filtered with a fourth-order bi-directional Butterworth bandpass filter with a cutoff frequency of 1–20 Hz [28] and triaxial gyroscope data were subjected to the same bandpass filter with a different cutoff frequency (0.25–30 Hz) [29]. The data collected from the distal tibia (shank) IMU sensor in this study were used to define the left and right gait cycle independently for each side. 

After being filtered with the bandpass filter with the cutoff frequency at 0.25–30 Hz, the mediolateral axis of the gyroscope data was used to detect initial contact (IC) and final contact (FC) of the left and right gait cycle [5,29]. The local maximum of the filtered shank angular velocity was selected as the mid-swing area, and additional criteria were defined for peaks larger than 50°/s to prevent false peak selection. After the mid-swing area had been defined, other local minima before and after the mid-swing event were selected to be the FC and IC events, respectively. Specified minimal peak distance and minimal peak height were also applied to IC and FC peak detection to prevent the system from detecting the wrong peak.

The resulting IC and FC event times formed a complete gait cycle with the following sequence: IC right foot → FC left foot → IC left foot → FC right foot → IC right foot → FC left foot → IC left foot. This gait cycle comprised two complete stride cycles of the right and left feet (IC to the next ipsilateral IC gait event). Gait temporal parameters were calculated from this complete gait cycle.

Stride time:

(1) $$Stride\;Time=IC_{\left(k+1\right)}-IC_{k}.$$

Stance time:

(2) $$Stance\;Time=FC_{\left(k\right)}-IC_{k}.$$

Stance time:

(3) $$Swing\;Time=IC_{\left(k+1\right)}-FC_{k}.$$

First double-limb support:

(4) $$1\mathrm{st}\;DLS=FC_{\left(k\right)contralateral}-IC_{\left(k\right)ipsilateral}.$$

Second double-limb support:

(5) $$2\mathrm{nd}\;DLS=FC_{\left(k\right)ipsilateral}-IC_{\left(k\right)contralateral}.$$

where *k* is the *k*th-order gait event.

The percentage of each gait temporal parameter was also calculated by dividing it by the stride time of its side of the gait cycle. The symmetry ratio between the left and right sides was also calculated for stride, stance, and swing parameters [30].

(6) $$Symmetry\;Ratio=\frac{Right\;temporal\;gait\;parameter}{Left\;temporal\;gait\;parameter}$$

In addition to calculating gait temporal parameters, the IC and FC event times were also used to separate the accelerometer and gyroscope data into each left and right gait cycle. Data from both the triaxial accelerometer and gyroscope were segmented into the left and right strides (IC to the next ipsilateral IC gait event) and then normalized into 101 points so that all segmented data would be the same size. After the time normalization process, time domain features were extracted from the normalized data and saved for further classification purposes.

The following time domain features were extracted from the accelerometer and gyroscope data: mean, variance, kurtosis, and deviation from the normalized segmented data. Although the time domain features were extracted from all seven sensors, cycle cutting was only applied to the corresponding side (for example, the left thigh sensor was applied to the cycle from the left stride and vice versa). Therefore, a total of 192 time domain features were extracted from all seven sensors for each trial on each subject. All trials for every subject could then be combined for use in further classification processing.

### 2.5. Gait Event Detection

**Figure 2:**
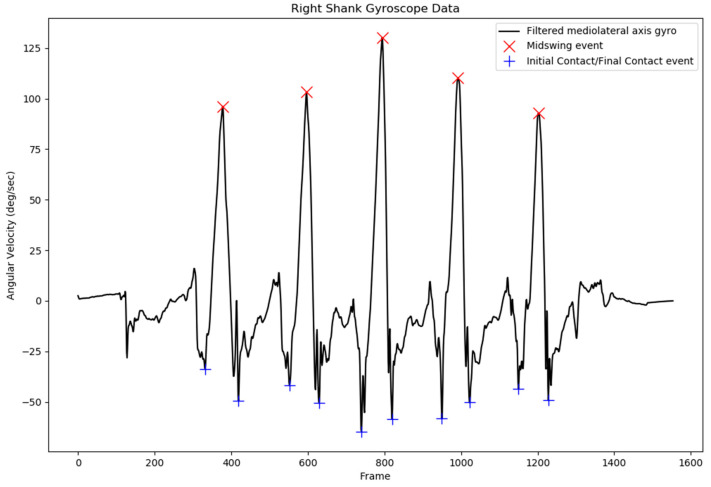
Initial contact (IC) and final contact (FC) event time detection from the filtered right-shank angular velocity signal.

The gait event consisting of IC and FC times was extracted from the shank angular velocity data using the described method. The result of IC and FC event time detection on the right side of the gait cycle is presented in Figure 2. The local minimum before the mid-swing event was the FC event time, and the local minimum after the mid-swing was the IC event time.

After several configurations, specified values of minimal peak distance and minimal peak height were set at 30 data points and 100°/s for the mid-swing event, respectively. For local minima point detection, minimal peak distance and minimal peak height were set to be 50 data points and 30°/s, respectively. These values were selected to prevent detection of the wrong IC and FC event times.

**Figure 3:**
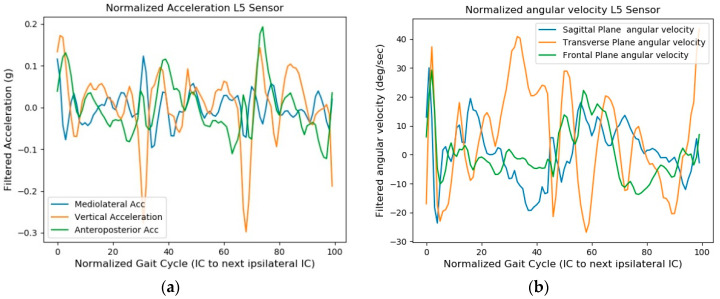
Normalized segmented acceleration (**a**) and angular velocity (**b**) obtained from the L5 IMU sensor data for feature extraction purposes.

The defined IC and FC event times were used to segment the filtered accelerometer and gyroscope signals into one gait cycle, for which the defined starting and end points were the IC event time and the next ipsilateral IC event time, respectively. Data for the normalized segmented acceleration and angular velocity are displayed in Figure 3.

### 2.6. Classification

Binary classification to differentiate stroke and other neurological disorders was performed using time domain features of the segmented accelerometer, gyroscope, and gait temporal features. Seven classification methods were used in this study, and the different configurations of sensors were also used to examine the most favorable sensor placement for distinguishing between the different groups. The five algorithms used in this study were as follows:

- Random forest (RF) classifier: A random forest is a supervised learning algorithm that combines decision trees and generally involves training with the bagging method. The number of trees used in this study was 1000.
- Adaboost classifier: This method was first introduced by Freund and Schapire [31]. The core principle of Adaboost is to fit a sequence of weak learners on repeatedly modified versions of data. In this study, the maximum number of estimators—at which boosting was terminated—was set to 150.
- Decision tree (DT): A decision tree is a tree-like graph that illustrates all possible decision alternatives and corresponding outcomes. It is composed of nodes where tests on specific attributes have been performed as well as leaf nodes indicating the value of the target attribute.
- Gaussian naïve Bayes (GNB): GNB is a supervised learning method that is based on applying the Bayes theorem with the *naïve* assumption of independence between each pair of features [32].
- Neural network with multilayer perceptron (MLP): MLP is a neural network capable of understanding a rich variety of nonlinear problems and uses backpropagation to classify instances [33]. The size of the hidden layer used in this study was 2000, with the *Adam* solver and an adaptive learning rate.

Hidden Markov Model (HMM)-based features and SVM classifiers were excluded in this study since these two algorithms have already been explored in many studies which classify either different types of gait patterns or different types of physical activity with wearable accelerometer/IMU sensors [12,22,34,35].

The time domain features of all subjects were combined with their corresponding true label to shape the whole subject dataset. The whole subject dataset was then split randomly with a 6:4 ratio into the training and testing datasets, respectively. The training dataset was then split 5-fold to perform *k*-fold cross-validation. The training dataset was trained with those five different algorithms and the mean accuracy of the 5-fold cross-validation method was calculated for each model. The trained model was tested with the unseen testing dataset, and the confusion matrix and accuracy were calculated and compared for each model.

In addition to the feature classification of all sensors, another classification was performed in which only the separate locations of each sensor were used as the feature with the five algorithms. The results from the classification of each sensor were then compared to identify the sensor with the best placement configuration for differentiating between the three groups.

Another classification method with calculated gait temporal parameters as the feature was used with the five algorithms. All 17 gait temporal parameters were used as the feature for the five algorithms, and the cross-validation and testing accuracies were reported and compared for each algorithm.

The next classification method combined the time domain features from the accelerometer and gyroscope with the calculated gait temporal parameters to acquire a new feature set. The new feature set then trained various algorithms to obtain the cross-validation and testing accuracy for each algorithm.

**Figure 4:**
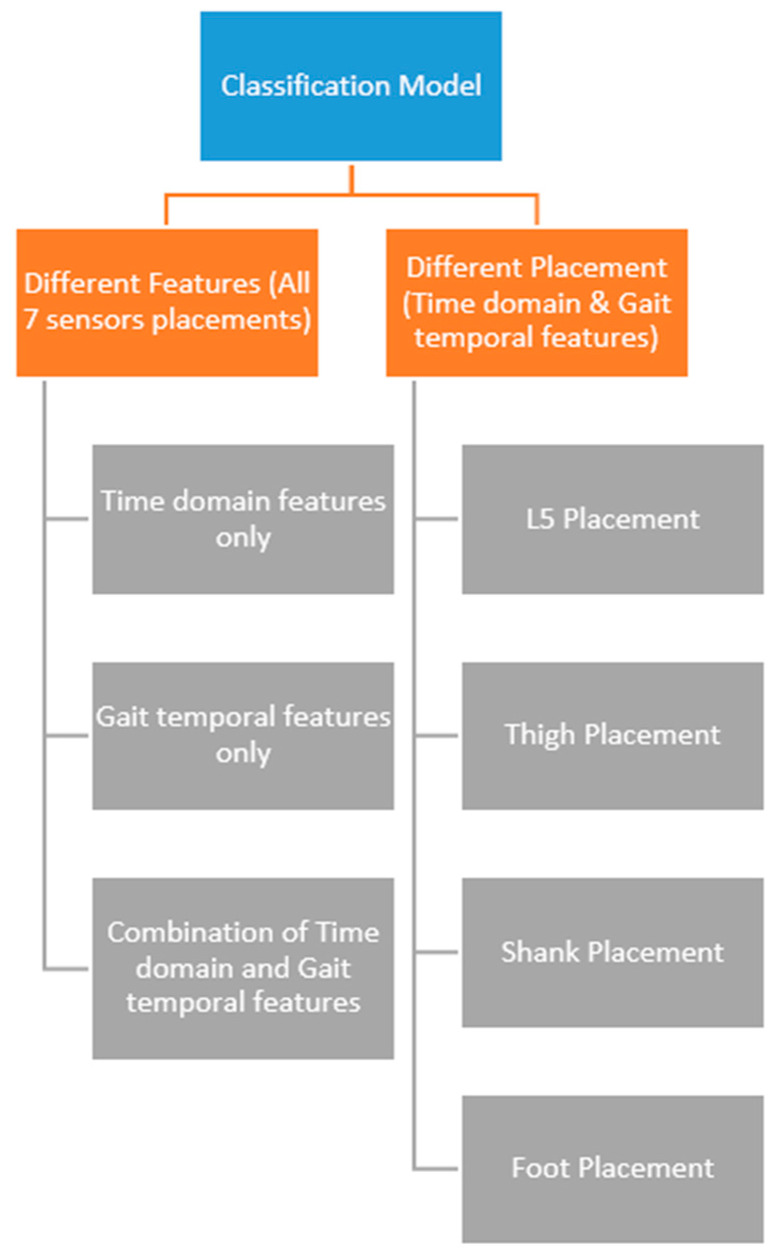
Chart of the seven different classification methods used in this study; the left part shows the three classification methods that used all sensor placements while the right part shows the four classification methods that used each sensor placement independently.

The last classification method used time domain and gait temporal features from each sensor location group separately for each independent classification. For example, the time domain features from the accelerometer and gyroscope on the left and right feet were combined to produce an independent classification result for the foot sensor. The same rule was also applied for the thigh, shank, and L5 sensors to produce four classification results for the foot, shank, thigh, and L5 sensor locations. A chart illustrating the combination of classification methods used in this study is shown in Figure 4.

To eliminate redundancy and reduce the number of features, feature selection was performed according to the highest score. In this study, the feature selection method employed was a calculation of the ANOVA F-value between the label and feature, and we selected only the highest-scoring 20% of features. The new feature set resulting from reduction through the feature selection process was used in the same classification method. All feature selection processes and classifications were performed with the *scikit-learn 0.19.1* module of Python 3.6.

## 3. Results

Time domain features were extracted from the normalized segmented data for the triaxial accelerometer and gyroscope at the seven sensor locations. The features were then used as the input for the five classifiers, the results of which are presented in Table 1.

The results obtained after the feature selection process are listed in Table 1. These results were derived from the reduced number of features, specifically only the 20% highest-scoring features according to the ANOVA F-values. The result of selected features from all the classification method used on this study showed on Appendix A.

A second classification experiment was conducted using the features for calculating gait temporal parameters. All 17 gait temporal parameters were used as features. The mean accuracy of the 5-fold cross-validation and the testing accuracy are presented in Table 2.

Table 3 presents the results from the classification experiment that used a combination of temporal gait parameters and time domain features with all seven sensor placements. The results from the previous classification experiment, which produced four results from four different sensor locations (L5, foot, shank, and thigh), are listed in Table 4, Table 5, Table 6 and Table 7. Cross-validation mean accuracy and testing accuracy after the feature selection process are also indicated in the results.

## 4. Discussion and Future Works

### 4.1. Discussion

This study aimed to perform a comprehensive analysis of multiple placements of wearable sensors for gait analysis and classification in patients with neurological disorders. Seven IMU sensors were placed at different locations, and gait-cycle-segmented data were used to create time domain features for classification purposes. The IC and FC gait event times were determined using filtered mediolateral acceleration data from the shank sensor. This algorithm has been commonly applied in studies using IMU sensors to define IC and FC gait event times, both in healthy individuals and in those with pathological disorders [5], including application for patients with knee arthroplasty [36], spinal cord injuries [37], and Parkinson’s disease [29]. All of these studies have been in agreement and confirmed the validity of using the shank angular velocity to define gait events with the golden standard, such as motion capture systems or foot switches. De Vroey et al. (2018) [36] reported adequate to excellent intra-class correlation values overall for temporal gait parameters calculated using shank angular velocity and a camera system, also suggesting that IMU sensors can be used outside of laboratory assessments to examine the temporal gait parameters in the knee arthroplasty population.

Because this event detection method was crucial for separating the accelerometer and gyroscope data for classification purposes in the present study, the use of IMU sensors to define the IC and FC events in the study that validated the method was therefore imperative. Thus, the study conducted by K. Aminian et al. [5] was significant for the conduction of the present study because it validated the algorithm for defining IC and FC events using shank angular velocity in healthy young and elderly subjects. The results also revealed high acceptability in elderly subjects and the method was recommended for use in clinical applications, such as the monitoring of rehabilitation progress, gait analysis in patients with neurological disorders, and fall risk assessment in elderly patients.

**Figure 5:**
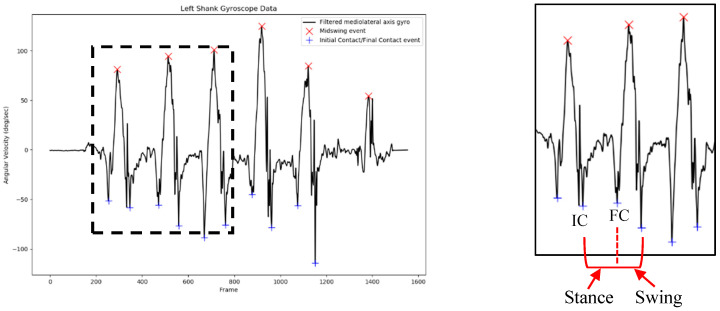
Typical shank angular velocity data from the nonaffected side of a stroke subject and the detailed IC and FC event times (right side).

**Figure 6:**
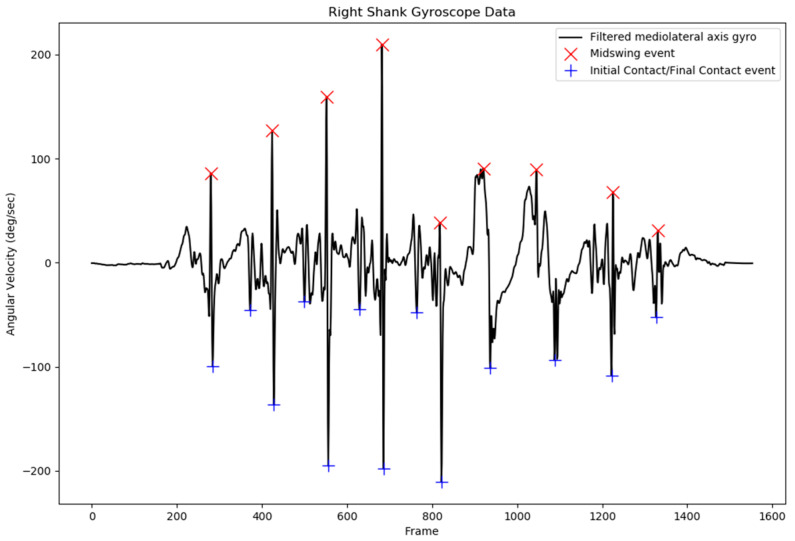
Typical shank angular velocity data from the affected side of stroke subjects. The figure indicates more abrupt and noisy signals in comparison with the nonaffected side.

The results from the IC and FC event detection revealed that false peak detection, which can lead to false IC and FC event detection, can be prevented with the current additional parameters applied for peak detection. The pattern in the shank angular velocity of the nonaffected side of stroke subjects was similar to that of the shank angular velocity of healthy subjects, with a less abrupt signal, as indicated in Figure 5. Although the shank angular velocity pattern of the affected side of stroke patients exhibited a more abrupt signal, the peak was still detected without any false peak detection. This phenomenon was also observed in another study involving a patient with a spinal cord injury; however, the study concluded that the gait event from the shank angular velocity could be used to detect the IC and FC events as accurately as a foot switch for both healthy subjects and those with pathological disorders [37]. An example of the shank angular velocity of a stroke subject’s affected side is presented in Figure 6.

The IC and FC event detections were used to separate the acceleration and angular velocity data from the seven sensor positions for gait classification. This study performed several classification experiments using features from the time domain, gait temporal parameters, and a combination of time domain and gait temporal parameters. Classifications using only independent sensor locations with time domain and gait temporal features were also performed to examine the most accurate sensor placements for gait classification.

The cross-validation accuracy and testing accuracy after feature selection were compared between the classification experiment and within-classification experiment (for different classification algorithms). The results revealed that for classifications involving only time domain features, the Naïve Bayes and MLP classifier outperformed other algorithms with 84.78% testing accuracy; however, MLP had better average precision at 0.86. Moreover, when the features changed from time domain to gait temporal parameters, the RF classifier achieved the highest testing accuracy (76.08%).

The third classification experiment combined time domain features and gait temporal parameters, and the results revealed that the MLP classifier outperformed other algorithms with 84.78% testing accuracy; MLP also had better average precision at 0.88. The results from the first three classification experiments, which used different types of features on the same sensor placement configuration, revealed that the classification with a combination of time domain and gait temporal features showed the best result with 84.78% testing accuracy and average precision and recall of 0.88 and 0.85, respectively. This testing accuracy was achieved with the MLP algorithm.

Classification with only gait temporal parameters demonstrated the lowest testing accuracy (76.08%) (with the RF method), in comparison with a combination of time domain and gait temporal features (84.78% testing accuracy with the MLP algorithm) or even with only time domain feature classification. Other studies have also reported that time domain and frequency domain features achieved higher accuracy than other classification methods using group-specific features based on the Hidden Markov Model (HMM) [12]. Although these studies used a classification algorithm (SVM) not used in the present study, the comparison of classification algorithms used in this study indicated that even using the same algorithm, the classification methods based on time domain features still outperformed those using gait temporal features and a combination of features (such as the RF method in this study). Moreover, an SVM classifier was already explored in a study classifying healthy young and older gaits [34] and in another classifying gaits between normal, hemiplegia, and PD patients [35] with both of them showing good results. Combined with HMM-based features, an SVM classifier was also explored in two previous studies by Mannini et al. which classified gaits between elderly, post-stroke, and Huntington’s disease subjects [12] and classified human physical activity [22]. However, these results are in agreement with those reported in the present study because the classification results from a combination of features were more favorable than the results from using only gait temporal parameters.

Another study also used time domain features and gait temporal parameters and discovered that their method was capable of classifying three types of neurodegenerative diseases (Parkinson’s disease, Huntington’s disease, and amyotrophic lateral sclerosis) with up to 90.63% accuracy [38]. In contrast with our results, this study reported relatively higher accuracy for the features extracted from gait temporal parameters. The fact that both the method utilized for defining the IC and FC events and the classification algorithm differed from those used in the present study may be a reason as to why their study achieved higher accuracy. Moreover, their study extracted the time domain features from gait temporal parameters, which also influenced their results. This study also found that among the set of all features, the gait temporal parameters feature was shown to have the worst classification performance with the lowest testing accuracy (60.8%). That means that the classification which only used gait temporal parameters was able to differentiate those groups but the performance was far worse than those of other classifications which utilized the other feature sets like time domain features from the acceleration and gyroscope.

The second part of the classification experiment was a comparison of classification using different sensor placements using the same type of features (combination of time domain and gait temporal features). In this part, four sensor placement groups (L5, foot, shank, and thigh) were used for different independent classification experiments. For the foot, shank, and thigh, the features used were derived from sensors on the left and right side. The results revealed that the shank-based placement achieved the highest result with 89.13% testing accuracy with the Decision Tree algorithm. This result was in agreement with the authors’ hypothesis which stated that shank-based placement might have better accuracy since the gait event definition was based on the shank sensor. Moreover, shank-based placement also outperformed all of the classification experiments which used all sensors.

These results could be attributable to the fact that all of the definitions of gait event were based on the angular velocity of the shank sensor, leading to the classification results based on the shank and thigh sensors demonstrating the highest accuracy. The results from the highest accuracy, precision, and recall from each classification method and sensor placement are shown in Table 8.

Another study that also used the same algorithm to define the IC and FC gait events compared the configuration of various sensor placements for detecting IC and FC events and calculated the spatiotemporal parameters of gait in children with cerebral palsy (CP). Their results revealed that a shank-and-thigh sensor configuration yielded more robust results in children with CP and a higher level of disability [26]. This result is in agreement with our results, which revealed that the shank and thigh sensor placement group achieved the highest accuracy among the sensor placements; moreover, other results proved that the algorithm using shank angular velocity to define IC and FC gait event times is more robust than other sensor placement configurations. 

### 4.2. Study Limitations and Future Works

The features used in this study did not include frequency domain features which may also be useful features for classifying between normal and abnormal gait. However, the current result which used a combination of time domain and gait temporal parameters already showed good results with 89.13% testing accuracy. Therefore, future study will examine the effect of adding frequency domain features for gait impairment classification. The limited sample size was also a limitation of this study, in addition to the individual differences that might exist among stroke patients and other neurological disease patients.

Future works arising from this study will be clinical applications that use the best sensor placement method showed in this study’s results in order to reduce the number of sensors used and increase the subject’s convenience.

## 5. Conclusions

The results of IC and FC gait event detection using shank angular velocity demonstrated robust detection in all three groups. False peak detection, which can lead to false gait event detection, could be prevented by using this algorithm in combination with the correct additional parameters, such as specified minimal peak distance and minimal peak height. 

The comparison of various methods and features for classification among the three groups demonstrated that the classification which used features from a combination of time domain and gait temporal parameters outperformed the classification which only used features from gait temporal parameters. The best result from combination-based feature classification was achieved by the MLP algorithm with 84.78% testing accuracy.

Classification results from different sensor placements revealed that the shank-based sensor gives the best result among the sensor placements with a testing accuracy with 89.13%. The results of the present study demonstrated that the wearable IMU device is capable of differentiating between the gait patterns of healthy patients, patients with stroke, and patients with other neurological disorders.

In summary, the best classification model result among all features and sensor placement combinations was achieved by using all placements with a combination of time domain and gait temporal parameters, while the best sensor placement was shank-based sensors with a combination of time domain and gait temporal parameters and the DT algorithm. Meanwhile, the worst sensor placement resulted from the RF algorithm using L5-based sensor placement. 

## Figures and Tables

**Table 1:** Validation and testing accuracy results for the five classification methods that used time domain features of normalized segmented acceleration and angular velocity from the data from the seven sensor positions.

Seven Sensor Placements (Time Domain Features)					
Model Name	5-Fold Cross-Validation		Testing Accuracy (%)	Average Precision	Average Recall
	Mean Accuracy (%)	Standard Deviation			
Random Forest	81.08425	10.78727	82.6087	0.83	0.83
Multilayer Perceptron	78.41758	11.86194	84.78261	0.86	0.85
Naïve Bayes	86.90842	5.415183	84.78261	0.85	0.85
Adaboost	83.84615	10.60653	76.08696	0.81	0.80
Decision Tree	79.64103	10.87058	76.08696	0.76	0.76

**Table 2:** Validation and testing accuracy results for the five classification methods that used features from gait temporal parameters.

Seven Sensor Placements (Gait Temporal Features)					
Model Name	5-Fold Cross-Validation		Testing Accuracy (%)	Average Precision	Average Recall
	Mean Accuracy (%)	Standard Deviation			
Random Forest	58.95238	11.24712	76.08696	0.76	0.76
Multilayer Perceptron	68.15385	11.58815	63.04348	0.68	0.63
Naïve Bayes	68.98168	11.92694	71.73913	0.76	0.72
Adaboost	55.98535	11.97776	65.21739	0.68	0.67
Decision Tree	51.49451	14.74979	60.86957	0.61	0.61

**Table 3:** Validation and testing accuracy results for the five classification methods that used a combination of gait temporal parameters and time domain features.

Seven Sensor Placements (Combination of Time Domain & Gait Temporal Features)					
Model Name	5-Fold Cross-Validation		Testing Accuracy (%)	Average Precision	Average Recall
	Mean Accuracy (%)	Standard Deviation			
Random Forest	80.87912	11.62656	84.78261	0.83	0.83
Multilayer Perceptron	71.05495	13.12726	84.78261	0.88	0.85
Naïve Bayes	88.22711	3.997853	84.78261	0.85	0.85
Adaboost	79.75092	8.194112	82.6087	0.85	0.85
Decision Tree	78.1978	4.665143	80.43478	0.84	0.80

**Table 4:** Validation and testing accuracy for the classification method that used time domain and gait temporal features of the accelerometer and gyroscope at the L5 sensor location.

L5 Sensor Placement (Combination of Time Domain & Gait Temporal Features)					
Model Name	5-Fold Cross-Validation		Testing Accuracy (%)	Average Precision	Average Recall
	Mean Accuracy (%)	Standard Deviation			
Random Forest	66.43956	10.10788	80.43478	0.81	0.80
Multilayer Perceptron	65.68498	19.98486	50	0.68	0.61
Naïve Bayes	75.02564	10.49391	73.91304	0.81	0.78
Adaboost	66.21978	10.95326	80.43478	0.80	0.80
Decision Tree	63.56777	5.392539	65.21739	0.78	0.78

**Table 5:** Validation and testing accuracy for the classification method that used the time domain and gait temporal features of the accelerometer and gyroscope at the foot sensor location.

Foot Sensor Placement (Combination of Time Domain & Gait Temporal Features)					
Model Name	5-Fold Cross-Validation		Testing Accuracy (%)	Average Precision	Average Recall
	Mean Accuracy (%)	Standard Deviation			
Random Forest	70.31502	17.46668	82.6087	0.83	0.83
Multilayer Perceptron	56.60073	5.390239	60.86957	0.68	0.61
Naïve Bayes	79.01099	13.31621	78.26087	0.81	0.78
Adaboost	67.53846	12.53356	80.43478	0.80	0.80
Decision Tree	67.45788	15.14377	78.26087	0.78	0.78

**Table 6:** Validation and testing accuracy for the classification method that used the time domain and gait temporal features of the accelerometer and gyroscope at the shank (distal tibia) sensor location.

Shank Sensor Placement (Combination of Time Domain & Gait Temporal Features)					
Model Name	5-Fold Cross-Validation		Testing Accuracy (%)	Average Precision	Average Recall
	Mean Accuracy (%)	Standard Deviation			
Random Forest	81.0696	5.926482	82.6087	0.83	0.83
Multilayer Perceptron	81.27473	7.196452	80.43478	0.86	0.80
Naïve Bayes	83.94139	7.054515	82.6087	0.83	0.83
Adaboost	75.23077	4.176749	76.08696	0.76	0.76
Decision Tree	72.37363	5.568556	89.13043	0.90	0.89

**Table 7:** Validation and testing accuracy for the classification method that used the time domain and gait temporal features of the accelerometer and gyroscope at the thigh sensor location.

Thigh Sensor Placement (Combination of Time Domain & Gait Temporal Features)					
Model Name	5-Fold Cross-Validation		Testing Accuracy (%)	Average Precision	Average Recall
	Mean Accuracy (%)	Standard Deviation			
Random Forest	77.91209	13.57666	80.43478	0.83	0.83
Multilayer Perceptron	52.20513	1.810191	54.34783	0.30	0.54
Naïve Bayes	79.64103	5.478678	82.6087	0.85	0.83
Adaboost	69.39194	6.124936	80.43478	0.79	0.78
Decision Tree	64.08791	7.802167	69.56522	0.70	0.70

**Table 8:** Result from the highest accuracy, precision, and recall from each classification method and sensor placement.

Classification Method (Algorithm Used):	Testing Accuracy (%)	Average Precision	Average Recall
Shank-placement-feature-based model (DT)	89.13	0.90	0.89
Thigh-placement-feature-based model (RF)	80.43	0.83	0.83
Time-domain-feature-based model (MLP)	84.78	0.86	0.85
Model based on gait temporal parameter features (RF)	76.08	0.76	0.76
Combination-feature-based model (MLP)	84.78	0.88	0.85

## Appendix A

In this section, the selected features from the feature selection process are shown for each classification model (Table A1).

**Table A1:** Result of selected features from each classification model.

Model Name	Result of Selected Features
Time domain features only	Variance of left foot acceleration Z, Mean of left foot acceleration Y, Mean of left shank acceleration X, Mean and kurtosis of left thigh acceleration X, Skewness of left shank acceleration Z, Skewness of left thigh acceleration X, Variance of right foot acceleration X, Mean of right foot acceleration Z, Kurtosis of L5 acceleration X, Kurtosis of right thigh acceleration X, Skewness of right foot acceleration X, Skewness of right thigh acceleration X, Variance of left foot gyroscope X, Y, and Z, Variance of left shank gyroscope X and Y, Variance of left thigh gyroscope Y, Kurtosis of L5 gyroscope Z, Kurtosis of left shank gyroscope X, Skewness of left foot gyroscope Y, Skewness of left thigh gyroscope Y and Z, Variance of right shank gyroscope X, Y, and Z, Variance of right thigh gyroscope X and Y, Mean of right foot gyroscope Y, Mean of right shank gyroscope Y, Kurtosis of right foot gyroscope X and Y, Kurtosis of right shank gyroscope X, Kurtosis of right thigh gyroscope Z, Skewness of right foot gyro X and Z, Skewness of right shank gyroscope X and Y.
Gait temporal features only	Right stance percentage, Right swing time, Right swing percentage, Swing ratio
Combination features (gait temporal and time domain frequency)	Right stance percentage, Right swing time, Right swing percentage, Swing ratio, Variance of left foot acceleration Z, Mean of left foot acceleration Y, Mean of left shank acceleration X, Mean and kurtosis of left thigh acceleration X, Skewness of left shank acceleration Z, Skewness of left thigh acceleration X, Variance of right foot acceleration X, Mean of right foot acceleration Z, Kurtosis of L5 acceleration X, Kurtosis of right thigh acceleration X, Skewness of right foot acceleration X, Skewness of right thigh acceleration X, Variance of left foot gyroscope X, Y, and Z, Variance of left shank gyroscope X and Y, Variance of left thigh gyroscope Y, Kurtosis of L5 gyroscope Z, Kurtosis of left shank gyroscope X, Skewness of left foot gyroscope Y, Skewness of left thigh gyroscope Y and Z, Variance of right shank gyroscope X, Y, and Z, Variance of right thigh gyroscope X and Y, Mean of right foot gyroscope Y, Mean of right shank gyroscope Y, Kurtosis of right foot gyroscope X and Y, Kurtosis of right shank gyroscope X, Kurtosis of right thigh gyroscope Z, Skewness of right foot gyro X and Z, Skewness of right shank gyroscope X and Y.
L5 sensor placement	Variance of gyroscope X and Z, Kurtosis of gyroscope Z, Mean of acceleration Z, Kurtosis of acceleration X, Right stride time, Right stance time, Right swing time and percentage, First Double Limb Support time and percentage, Stride and Swing ratio.
Foot sensor placement	Variance of left foot gyroscope Y, Skewness of left foot gyroscope Y, Variance of right foot acceleration X, Mean of right foot acceleration Z, Skewness of right foot acceleration X, Kurtosis of right foot gyroscope X, Mean of right foot gyroscope Y, Skewness of right foot gyroscope Z, Right stance percentage, Right swing time and percentage, Stride and Swing ratio.
Shank sensor placement	Skewness of left shank acceleration Z, Variance of left shank gyroscope X, Variance of right shank gyroscope X and Y Mean of right shank gyroscope Y, Kurtosis of right shank gyroscope X, Skewness of right shank gyroscope X and Y, Right stance percentage, Right swing time and percentage, Stride and Swing ratio.
Thigh sensor placement	Variance of left thigh gyroscope Y, Skewness of left thigh gyroscope Y, Skewness of right thigh acceleration X, Variance of right thigh gyroscope X and Y, Kurtosis of right thigh gyroscope Z, Right stance percentage, Right swing time and percentage, Stride and Swing ratio.
